# Magnetic Resonance Imaging-Based Assessment of Bone Marrow Fat and T2 Relaxation in Adolescents with Obesity and Liver Steatosis: A Feasibility Pilot Study

**DOI:** 10.3390/jcm14217594

**Published:** 2025-10-26

**Authors:** Camille Letissier, Kenza El Ghomari, Sylvie Gervais, Léna Ahmarani, Ramy El Jalbout

**Affiliations:** 1 Department of Medical Imaging, CHU Sainte-Justine Hospital, Faculty of Medicine, University of Montreal, Montreal, QC H3T 1C5, Canada; kenza.el.ghomari@umontreal.ca (K.E.G.); lena.ahmarani.hsj@ssss.gouv.qc.ca (L.A.); ramy.el-jalbout.med@ssss.gouv.qc.ca (R.E.J.); 2 Département de Génie Logiciel, École de Technologie Supérieure (ÉTS), Montreal, QC H3C 1K3, Canada; sylvie.gervais@etsmtl.ca

**Keywords:** bone marrow, adipose tissue, obesity, nonalcoholic fatty liver disease, dual-energy X-ray absorptiometry, bone density, adolescent

## Abstract

**Background:** Adolescents suffering from obesity are at higher risk of bone fragility due to hepatic steatosis, which may lead to an inflammatory microenvironment in the bone marrow. We therefore aimed to assess the reliability of measuring the bone marrow fat fraction (BMFF) and T2* of the lumbar vertebral marrow using the proton density fat fraction (PDFF) sequence for adolescents with obesity and liver steatosis. **Method:** This was an observational feasibility pilot study on adolescents living with obesity and liver steatosis. Anthropometric measurements were obtained. Participants underwent abdominal MRI, MR elastography and dual-energy X-ray absorptiometry (DXA). Regions of interest were drawn using the radiology interface from the central L1 to L4 vertebrae on fat and T2* maps from the PDFF sequence. ImageJ was used to measure abdominal compartment fat areas. Descriptive analyses, the intraclass correlation coefficient, and correlation results were obtained from anthropometric, adiposity, BMFF, and T2* measurements. **Results:** We recruited 23 adolescents with a body mass index > 85th percentile and mean age = 14.7 years (interval 12–17 years), and *n* = 18 (78%) were boys. BMFF and T2* measurements were successful in 100% of cases. The intra-operator reproducibility of the BMFF and T2* measurements was excellent: ICC = 0.99 (95% confidence interval (CI) [0.986; 0.999]) and ICC = 0.99 (95% CI [0.992; 0.999]), respectively. The inter-operator ICC was good for BMFF (ICC = 0.89; 95% CI [0.705; 0.963]) and moderate for T2* (ICC = 0.66; 95% CI [0.239; 0.873]). Only BMFF was inversely correlated with vertebral-bone mineral density (*r* = −0.67; *p* = 0.0009). However, T2* measurements showed a positive linear relationship with the total body fat tissue percentage measured by DXA (*r* = 0.48; *p* = 0.03) and the total abdominal fat area (*r* = 0.45; *p* = 0.04). **Conclusions:** PDFF could be a reliable imaging biomarker for bone health assessment in adolescents living with obesity.

## 1. Introduction

Overweight and obesity in childhood and adolescence is a growing worldwide health issue associated with increased morbidity and mortality [1,2].

One third of Canadians aged between 5 and 17 years live with overweight or obesity [3], and the prevalence of metabolic dysfunction-associated steatotic liver disease (MASLD) in the pediatric general population ranges from 9% to 37% [4].

Current evidence suggests that crosstalk occurs between the liver and bone marrow milieu and that hepatic steatosis, one of the major outcomes of obesity, might promote an inflammatory microenvironment in the bone marrow, directing the differentiation of mesenchymal cells toward adipocytes [5,6]. This could increase the bone marrow fat fraction (BMFF) at the expense of osteoblasts, ultimately causing lower bone mineral density (BMD) [7,8,9,10,11].

Thus, children and adolescents with obesity are at greater risk of developing bone fragility and experiencing pathological fractures, not to mention the economic impact [12,13]. Currently, the detection of osteoporosis is based on the gold standard, dual-energy X-ray absorptiometry (DXA), which allows for the evaluation of BMD by providing areal bone mineral density (g/cm^2^) [14]. However, there are some limitations to the use of this modality. One of the main disadvantages of DXA is its lack of performance for regional assessment of body composition, especially when the patient is suffering from obesity. Indeed, DXA precision declines with increasing body mass index (BMI) [15]. Second, DXA provides a 2D image of a 3D structure; thus, BMD overestimates true bone density in taller children with larger bones while underestimating it in shorter children with smaller bones. Since children living with obesity tend to have larger bones, the accuracy of DXA measurements is reduced [16].

Obesity is a low-grade systemic inflammatory disease that may increase iron tissue storage [17,18]. In fact, iron homoeostasis is regulated by hepcidin, a peptide hormone produced by hepatocytes. It is released, among other factors, in response to inflammation and induces iron storage.

Many studies have shown the utility of magnetic resonance imaging (MRI)-estimated proton density fat fraction (PDFF) sequences for the identification and quantification of bone marrow adipose tissue in vertebrae and their inverse correlation with BMD [11,19,20,21,22]. Indeed MRI could be an interesting complementary tool to DXA to evaluate bone health by providing 3D information and quantifying fat and iron using novel quantitative sequences. The resolution and performance of MRI could provide more precise information for potential further osteoporosis prevention treatment. These studies suggest that the vertebral fat fraction and T2* (reflecting iron stores) could be used as biomarkers for the screening of bone health. However, for the most part, these metrics have been examined in a healthy adult population or diagnosed with osteoporosis, and DXA has not been used as a reference standard. The use of quantitative MRI in children living with obesity who are otherwise healthy is scarce. Labayen et al. [7] studied the relationship between the lumbar spine bone marrow fat fraction and BMD using DXA in a pediatric population. However in the pediatric radiological literature, no studies have evaluated the T2* variable or its association with liver stiffness and BMFF.

We hypothesize that BMFF and T2* measurements are feasible and reproducible and correlate with BMD measured through the current gold standard, DXA, in children with obesity and liver steatosis. The main objective of this study is to assess the reliability of measuring the BMFF and T2* of the lumbar vertebral marrow using PDFF sequences.

## 2. Materials and Methods

### 2.1. Study Design

This is an observational prospective pilot study approved by Health Canada and the hospital’s ethics board, under ethics committee number 20202278, with assent and consent obtained from participants and their guardians. It is a sub-analysis of a prospective open-label randomized control study (ClinicalTrials.gov ID: NCT03994029) that included three visits scheduled at 8-week intervals, the protocol of which included a monitoring procedure performed during follow-up, with the results published in an open access journal [23].

### 2.2. Participants and Eligibility Criteria

**Figure 1:**
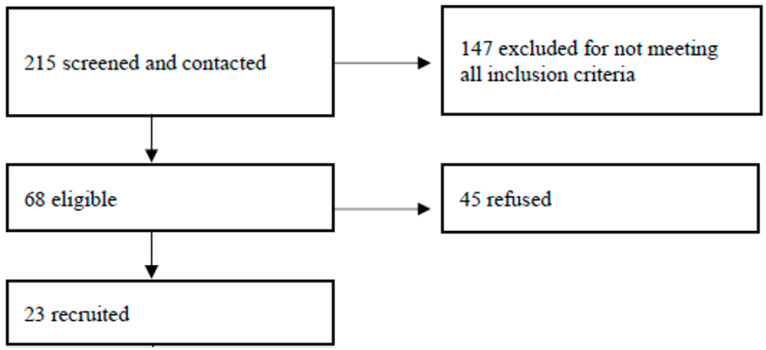
Flowchart for recruitment. A total of 23 participants were recruited for our study. As each participant underwent three visits, a total of 54 MRI scans with the full protocol were performed.

The participants were recruited from July 2021 to December 2024 from a list of patients who were being followed up at the hepatology liver steatosis clinic of the hospital. Figure 1 shows the flowchart for recruitment. Eligibility criteria included adolescents aged 12–18 years, with a BMI >85th percentile for age and sex and a diagnosis of hepatic steatosis on imaging (ultrasound or MRI), a diagnosis of hepatic steatosis, nonalcoholic steatohepatitis, or fibrosis on liver biopsy, an elevated alanine aminotransferase (ALT) enzyme level, or an index of hepatic steatosis 8 × ALT/aspartate aminotransferase (AST) + BMI (+2 for girls) > 30 [23]. Exclusion criteria included chronic systemic diseases or any other serious conditions, pregnancy, weight loss of 5% to 10% in the 6 months before recruitment or weight change of 5% in the last 3 months, liver fat < 5.5% on MR spectroscopy, alcohol consumption, and any contraindications for MRI.

### 2.3. Data Collection and Anthropometrics

Each visit involved anthropometric data assessed by qualified personnel: weight in kg, height in cm, BMI in kg/m^2^, waist circumference, hip circumference, calf circumference (each in cm), tricipital circumference, and infra-scapular and supra-iliac subcutaneous fold thicknesses (each in mm). Tanner’s stage was specified by the participant through self-assessment using a scientific illustration. Abdominal MRI and DXA were conducted, and liver biopsy results (from the prior year, if available in the participant’s hospital chart) were used for imaging comparisons.

### 2.4. Imaging Procedures

MRI scans were performed after 12 h of fasting using a 1.5 T Ingenia MRI system (Philips Healthcare, Best, The Netherlands) with an abdominal coil, with participants in a supine position and aided by a belt for free respiration.

#### 2.4.1. PDFF Sequence

**Figure 2:**
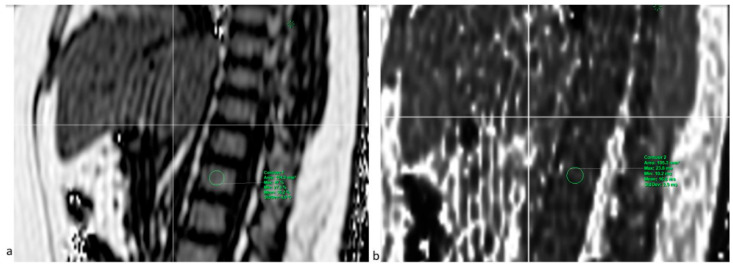
PDFF-mDixon-quant sequence of a 13.8-year-old male adolescent (BMI = 38.11 kg/m^2^). (**a**) Sagittal fat map with an ROI placed on L1 showing a BMFF of 35.2%; (**b**) sagittal T2* map with an ROI placed on the same vertebra, showing a T2* of 16.6 ms.

This MRI sequence (mDixon-quant) was used to measure the vertebral BMFF and T2*. Six echoes were used the first TE being as shown in Table 1. The combination of the anterior and posterior antennae gave a total of 28 coil elements. Images were extrapolated on Philips Intellispace Portal 12, and a region of interest (ROI) was placed in each of the L1 to L4 vertebral bodies on both fat and T2* maps (Figure 2). For reproducibility in observer-dependent measurements, a second observer independently collected the same measurements for the first 10 participants. This same sequence was used to measure the hepatic fat fraction for the quantification of hepatic steatosis.

#### 2.4.2. Liver Elastography

**Figure 3:**
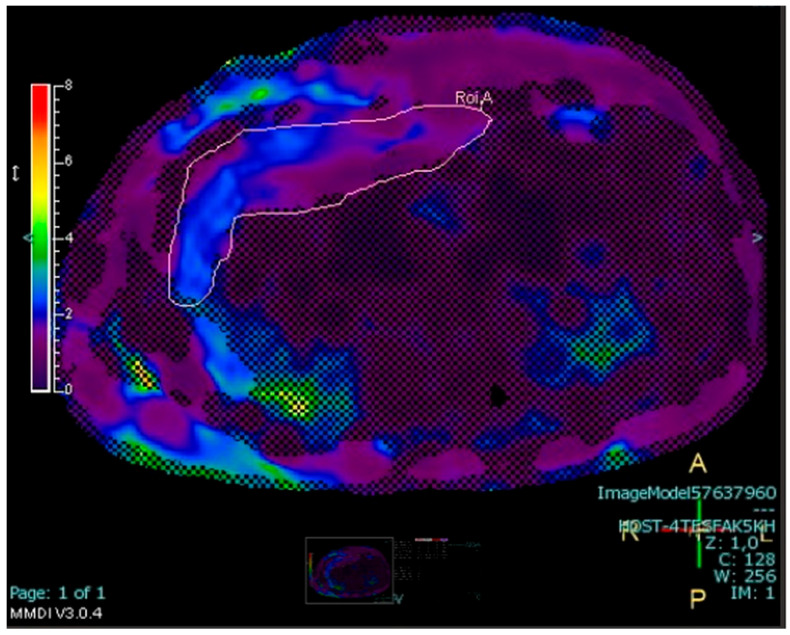
MR elastography of a 17.7-year-old male adolescent (BMI = 29.2 kg/m^2^) showing one of the four ROIs placed on the liver and automatically excluding areas of suboptimal wave propagation (shaded area). The average liver stiffness of the four ROIs for this patient was 1.8 kPa.

MR elastography was used to assess liver stiffness [24]. A Resoundant system with a pneumatic oscillator set to 60 Hz was connected to a Philips 1.5T Ingenia MRI system (Best, The Netherlands) to acquire a 2D gradient-recalled echo (GRE) sequence under breath hold, measuring four liver ROIs on different slices and automatically excluding areas of the liver showing suboptimal wave propagation (Figure 3).

#### 2.4.3. Measurement of Abdominal Fat Stores

Abdominal fat area measurement was performed on the same PDFF sequence in a single axial image in the midportion of the L2 vertebral body using ImageJ software (v1.53t 2022) [25]. The image was processed to isolate fat regions by setting a pixel threshold; then the desired ROIs were delimited. The measurements (in mm^2^) included total abdominal fat area—the combined fat within the abdominal wall and peritoneal cavity—and visceral fat (VAT) area—the fat enclosed by the abdominal wall muscles.

### 2.5. DXA Examination

The areal BMD (defined as grams per square centimeter of calcium hydroxyapatite) of cancellous bone in the whole body (total BMD) and spine (vertebral BMD) and the total body fat percentage (%total body fat) were measured by DXA (GE Lunar iDXA, GE Healthcare, Waukesha, WI, USA, serial number: 212689MA, Software Version 18), and the T score was recorded.

### 2.6. Statistical Analysis

All analyses were conducted with IBM SPSS Statistic software version 28.0.1.0 and Statgraphics software version 19.2.02 under biostatistician guidance (SG).

The statistical analyses included descriptive evaluations of anthropometric variables expressed as mean +/− standard deviation. Inter- and intra-observer reproducibility was verified using the intraclass correlation coefficient (ICC) for BMFF and T2* bone measurements. The association between BMFF, T2*, and anthropometric measurements was assessed via Pearson’s correlation coefficient. Correlation analyses were conducted between PDFF vertebral measurements (BMFF and T2*) and DXA and between PDFF vertebral measurements (BMFF and T2*) and abdominal MRI (hepatic steatosis, liver elastography, abdominal compartmental fat areas) using repeated-measures ANOVA. The potential differences in results among the three visits were compared through correlation for repeated measures. A *p* value less than 0.05 was considered statistically significant. Residual analysis was performed to evaluate the ANOVA and regression normality assumption, based on residual Q–Q plots, histograms, the Shapiro–Wilk test, and standardized skewness and kurtosis coefficients. If the normality assumption was violated, a Friedman test was used for the ANOVA and non-parametric regression methods were used for the regressions. No correction for multiple analyses was performed since this is an exploratory study.

## 3. Results

A total of 23 participants with liver steatosis were included in this study: 18 males (78.3%) aged 12–17 years (mean age: 14.8 ± 1.7 years). Table 2 shows the descriptive characteristics of the participants. Although the intended sample size was not achieved, the study design remains appropriate for meeting the study’s main objectives of feasibility and reproducibility.

Because the full MRI protocol was not always completed by all participants on all three visits, a total of 54 MRIs with PDFF (mDixon-quant) sequences were obtained. BMFF and T2* were measurable in 100% of cases when mDixon-quant was available, providing a feasibility of 100%.

The intra-observer reproducibility for BMFF and T2* measurements using the ICC test was excellent: ICC = 0.995 for BMFF (95% confidence interval (CI) [0.986; 0.999]) and ICC = 0.997 for T2* (95% CI [0.992; 0.999]). The inter-observer reproducibility was good for BMFF, with ICC = 0.890 (95% CI [0.705; 0.963]), and moderate for T2*, with ICC = 0.660 (95% CI [0.239; 0.873]).

The next statistical analyses were carried out based on the mean values of BMFF and bone marrow T2* values.

**Figure 4:**
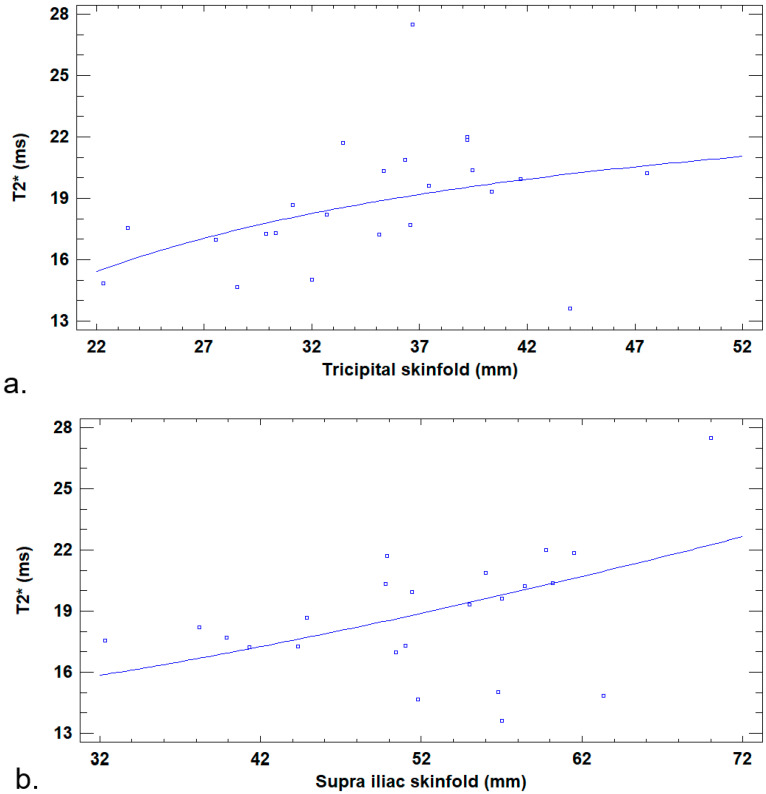
Nonlinear regression between bone marrow T2* and anthropometric values. (**a**) Graphic depiction of the adjusted model, with T2* = exp(3.274 − 11.829/tricipital skinfold), showing a nonlinear regression between T2* (y axis) and mean tricipital skinfold (x axis). (**b**) Graphical depiction of the adjusted model, with T2* = sqrt(186.487 + 0.063 × supra iliac skinfold), showing a nonlinear regression between T2* (y axis) and mean supra-iliac skinfold (x axis).

No significant linear correlation was found between the BMFF, bone marrow T2*, and anthropometric values. Two nonlinear positive regressions were found between the bone marrow T2* and mean tricipital skinfold values (R^2^ = 19%; *p* = 0.04) and between the bone marrow T2* and mean supra-iliac skinfold values (R^2^ = 22%; *p* = 0.02) (Figure 4).

**Figure 5:**
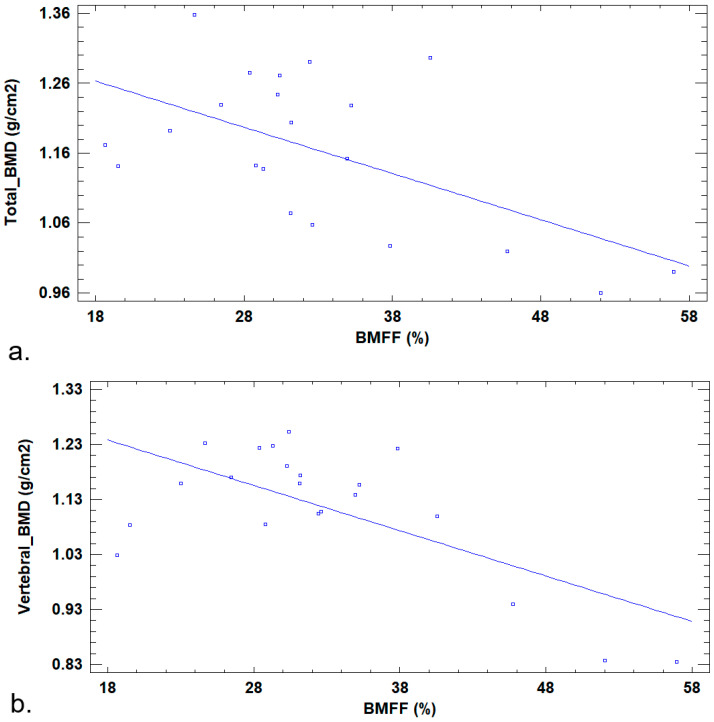
Linear regression between BMFF on MRI and BMD on DXA. The negative slope indicates a decrease in BMD as BMFF increases; that is to say that BMFF is inversely correlated with total BMD and vertebral BMD. This suggests that an increasing bone marrow fat fraction leads to reduced bone formation. (**a**) Linear regression between BMFF (x axis) and total BMD (y axis). Graphical depiction of the adjusted model, with Total_BMD = 1.382 − 0.006 × BMFF. (**b**) Linear regression between BMFF (x axis) and vertebral BMD (y axis). Graphical depiction of the adjusted model, with vertebral_BMD = 1.386 − 0.008 × BMFF.

BMFF was inversely correlated with total BMD (r = −0.58; *p* = 0.006) and with vertebral BMD (r = −0.67; *p* = 0.001) (Figure 5). No significant correlation was found between BMFF and %total body fat (r = 0.27; *p* = 0.23).

**Figure 6:**
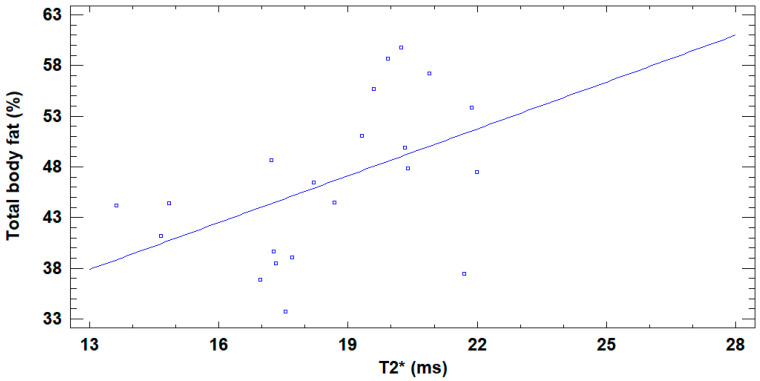
Linear regression between bone marrow T2* and %total body fat. Graphical depiction of the adjusted model, where %total body fat = 17.834 + 1.541 × T2*, showing the positive correlation between T2* (x axis) and %total body fat (y axis).

T2* was positively correlated with %total body fat (r = 0.48; *p* = 0.027) (Figure 6), whereas no significant correlation was found between T2* and total BMD (r = −0.11; *p* = 0.63) or between T2* and vertebral BMD (r = −0.02; *p* = 0.91).

No significant correlation was found between BMFF and hepatic steatosis (r = 0.08; *p* = 0.77) or between T2* and hepatic steatosis (r = −0.24; *p* = 0.36).

**Figure 7:**
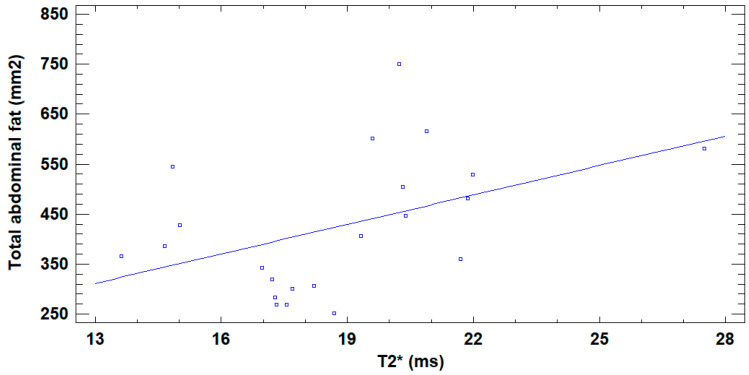
Linear regression between bone marrow T2* and total abdominal fat. Graphical depiction of the adjusted model, with total abdominal fat = 55.694 + 19.659 × T2*, showing the positive correlation between T2* (x axis) and total abdominal fat (y axis), as shown by the positive slope.

A positive correlation was found between bone marrow T2* and total abdominal fat (r = 0.45; *p* = 0.036) (Figure 7). No significant correlation was found between T2* and VAT (r = 0.09; *p* = 0.67), between BMFF and total abdominal fat (r = 0.02; *p* = 0.92), or between BMFF and VAT (r = −0.03; *p* = 0.89).

On MR elastography, 9% of participants had liver stiffness exceeding 2.77 kPa [26,27]. No significant correlation was found between BMFF and liver stiffness (r = 0.05; *p* = 0.80) or between bone marrow T2* and liver stiffness (r = 0.25; *p* = 0.25).

## 4. Discussion

The main objective of this study was to evaluate the feasibility, reliability, and reproducibility of quantitative MRI to measure bone marrow adipose deposition and iron stores in lumbar vertebrae in children with obesity. The measurement of BMFF and bone marrow T2* is feasible and reproducible on PDFF sequences in children with obesity. Inter-observer reproducibility was moderate for T2*, which can be easily explained by the fact that the T2* maps have low spatial resolution; thus, the limits between vertebral body bone marrow, cortical bone, and the intervertebral disk are sometimes more difficult to identify.

The secondary objective was to explore the correlation between the gold standard DXA and MRI measurements, as well as between the MRI data themselves. We found a negative relationship between BMFF and BMD, which is consistent with previous studies [7,8,11,20,22]. The correlation between bone marrow development and obesity has been detailed by several authors and relies on multiple mechanisms [28,29,30]. First, it has been proposed that obesity promotes an inflammatory microenvironment directing mesenchymal stem cell differentiation toward the adipocyte cell line at the expense of osteoblasts, resulting in reduced bone formation and an increased bone marrow fat fraction [31]. Second, obesity and bone marrow fat itself release pro-inflammatory molecules that will downregulate osteoblasts, osteocytes, and muscle cells and upregulate osteoclasts, causing an imbalance between osteoblastic and osteoclastic activities. Third, although we did not assess dietary intake, high-fat diets may reduce intestinal calcium absorption, causing decreased calcium availability for bone formation and boosting further inflammation. Moreover, hyperinsulinemia, which is commonly associated with obesity and MASLD, directly affects osteoblast–osteoclast pair, resulting in decreased bone remodeling and, consequently, impaired bone quality. Finally, physical activity is a major mechanical stimulus for bone accretion and is often reduced in children living with obesity [16].

The positive correlation between bone marrow vertebral T2*, total abdominal fat area, and %total body fat and the absence of a significant correlation between T2*, total BMD, and vertebral BMD can be explained by two opposing mechanisms. On one hand, systemic inflammation related to obesity is responsible for increasing iron tissue storage by activating hepcidin. Hepcidin is the main iron-regulating peptide hormone and is chiefly produced by hepatocytes in response to rising iron saturation of plasma transferrin, increased hepatic iron stores, or inflammation, and it induces negative feedback to reduce iron availability [17]. On the other hand, renal hypoxemia related to obesity and MASLD causes the kidneys to release erythropoietin, which stimulates erythropoiesis and the production of erythroferrone. Erythroferrone inhibits hepcidin release, thereby increasing intestinal iron absorption and the release of iron from stores. Our cohort of participants were found to have increased R2* (R2* = 1/T2*) values in their kidneys, reflecting kidney hypoxemia (data not yet published). Thus, the iron load in bone marrow (more iron is indicated by a lower T2* value) is dictated by the interaction between both mechanisms, the latter prevailing over the former in our cohort. This hypothesis could be verified in the future by measuring plasma iron levels.

We did not find any significant correlation between BMFF, bone marrow T2*, and hepatic steatosis, which is discordant with Labayen et al. In their study, children with hepatic steatosis had a significantly higher bone marrow fat fraction [7]. This might be explained by the small size of our population.

No significant correlation was found between BMFF and %total body fat or between BMFF, total abdominal fat, and VAT. The literature concerning a potential link between these variables is scarce. This result emphasizes that BMFF does not depend on the degree of obesity, which is also demonstrated by the fact that BMFF is increased in cohorts of patients suffering from osteoporosis with a normal BMI [32]. Moreover, it suggests a complexity in the mechanisms of adipogenesis and the distribution of adipocytes in the body. Similarly, this could explain the lack of a significant linear correlation between BMFF, bone marrow T2*, and anthropometric measurements, although mean tricipital skin thickness and mean supra-iliac skin thickness could be used as potential markers of bone marrow T2* in association with other parameters in a predictive model.

No significant correlation was found between BMFF, bone marrow T2*, and liver stiffness measured by MR elastography. It is difficult to draw conclusions from this result since only 9% of participants had increased liver stiffness.

Finally, the results emphasize the added value of MRI as a one-step exam to assess bone health, as other studies have also used MRI routinely for lower back pain as a screening tool for osteoporosis without any additional cost, radiation exposure, or time [33,34].

There are some limitations to this secondary objective. First, the small sample size might explain some unsignificant correlations that have previously been detailed.

The distribution between girls and boys could have been more balanced if the total number of participants had been higher. Therefore gender stratification of the results is not possible for our study. Second, inflammatory biomarkers and serum iron levels were not assessed in this cohort, which would have enabled an evaluation of systemic inflammation severity and verification of the hypothesis that serum iron levels are elevated in these children.

Thirdly, the duration of obesity could affect bone marrow fat and iron stores. This represents another limitation, as we could not obtain the duration of obesity for our cohort.

## 5. Conclusions

The current pilot study demonstrates the feasibility of using PDFF MRI to quantify bone marrow fat and iron content in the lumbar spine of adolescents living with obesity. Preliminary associations suggest that increased marrow fat may be associated with lower BMD and altered marrow iron content. However, we agree that the way our study is designed, along with its limitations, does not allow for such premature conclusions, and these findings require confirmation in larger, well-powered cohorts before clinical implications can be established. An expanded sample size, a better representation of both genders, a group of adolescents with obesity exhibiting abnormal DXA results, a control group with normal BMI and DXA, and the measurement of serum inflammatory markers and iron storage for each group would enhance the robustness of these preliminary findings.

## Figures and Tables

**Table 1:** Sequence parameters for MR elastography and PDFF.

	MR Elastography	PDFF
Sequence	MR Elastography-4SL FFE	mDixonQuant-BH FFE
FOV (mm)	400	400
Matrix	272 × 64	144 × 116
Number of slices	4	70
Slice thickness (mm)	10	6
TE (msec)	20	1.22
TR (msec)	50	6.7
Slice orientation	Transverse	Transverse
Gap (mm)	1	0
Flip angle (degree)	20	5
Time (seconds)	5 × 13	1 × 14
Parallel imaging	2 (SENSE)	
Inversion time	No	No
Breath hold	Yes (after expiration)	Yes

Abbreviations: PDFF, proton density fat fraction; FFE, fast-field echo; FOV, field of view; TE, echo time; TR, repetition time; SENSE, sensitivity encoding.

**Table 2:** Characteristics of participants.

Variables	Mean (Standard Deviation)
Age (years)	14.8 (interval 12–17)
BMI (kg/m^2^)	35.5 (±6.7)
Hepatic steatosis (%)	20.3 (±11.2)
Hepatic elastography on MRI (kPa)	2.2 (±0.5)
Total bone mineral density (g/cm^2^)	1.5 (±0.1)
Vertebral mineral density (g/cm^2^)	1.4 (± 0.1)
Total body fat (%)	45.3 (±7.3)

## Data Availability

Participant files will be maintained in storage for a period of 7 years after the completion of the study. The original research data is available upon request by email from the study’s principal investigator Ramy El Jalbout, email: ramy.el-jalbout.med@ssss.gouv.qc.ca.

## Abbreviations

The following abbreviations are used in this manuscript:

MASLD	Metabolic dysfunction-associated steatotic liver disease
BMFF	Bone marrow fat fraction
BMD	Bone mineral density
DXA	Dual-energy X-ray absorptiometry
BMI	Body mass index
MRI	Magnetic resonance imaging
PDFF	Proton density fat fraction
ALT	Alanine aminotransferase
AST	Aspartate aminotransferase
ROI	Region of interest
VAT	Visceral fat
ICC	Correlation coefficient
